# Determination Method of Optimal Decomposition Level of Discrete Wavelet Based on Joint Jarque–Bera Test and Combination Weighting Method

**DOI:** 10.3390/e27020108

**Published:** 2025-01-23

**Authors:** Zhanpeng Zhang, Changjian Liu, Min Wang, Shuang Sun, Zhao Zhan

**Affiliations:** 1 Key Laboratory of Smart Earth, Beijing 100080, China; zhanpeng1109@163.com (Z.Z.); different9@163.com (M.W.); daben64ss@163.com (S.S.); ndzdszzz@163.com (Z.Z.); 2 Institute of Geospatial Information, Information Engineering University, Zhengzhou 450001, China

**Keywords:** wavelet denoising, Gaussian white noise, Jarque–Bera test, comprehensive evaluation indicator, combined weighting, optimal decomposition level

## Abstract

To overcome the limitations of traditional evaluation indicators in determining the optimal wavelet decomposition level, this paper proposes an adaptive method for selecting the best decomposition level by combining the Jarque–Bera test and a composite weighting approach. Firstly, in the noise extraction stage, the Jarque–Bera test is employed to ensure that the extracted noise follows Gaussian white noise characteristics, thereby avoiding issues of insufficient denoising or signal distortion. Secondly, in the evaluation stage of the denoised signal, a comprehensive consideration of the geometric and physical meanings of various evaluation metrics, as well as the Pearson correlation coefficients between them, is undertaken. The RMSE and smoothness are selected as evaluation indicators for the denoising performance. Since these two metrics describe signal characteristics from different dimensions, a weighted combination approach is used to generate a single composite evaluation index. Additionally, to overcome the limitations of using a single weighting method, a composite weighting strategy is proposed by combining the entropy weight method and the coefficient of variation method. The composite coefficient between these two weighting methods is calculated using the variance coefficient method, yielding a new composite evaluation metric. A smaller value of this metric indicates better denoising performance, and the corresponding optimal decomposition level is more accurately determined. The simulation results demonstrate that the proposed comprehensive evaluation method can accurately determine the optimal wavelet decomposition level in both known and unknown truth-value cases, exhibiting a high accuracy and good applicability. Furthermore, the experimental results show that using the optimal decomposition level determined by the proposed method for wavelet denoising leads to smoother peak regions, more stable waveforms and significantly improved denoising performance.

## 1. Introduction

According to the Central Limit Theorem, under consistent measurement conditions, the superposition of multiple independent noise sources tends to result in a normal distribution, making it possible to model the noise in real-world measurements as Gaussian white noise for analysis [1,2].

In recent years, wavelet analysis, with its excellent time–frequency localization, multi-resolution capabilities, and ability to handle nonlinear problems, has been extensively applied in areas such as geodetic data denoising [3,4]. By processing signals using wavelet transform, noise interference can be effectively reduced, thus improving the accuracy and reliability of the results. However, the effectiveness of wavelet denoising largely depends on the appropriate choice of decomposition levels, as an improper selection may result in insufficient denoising or signal distortion. Therefore, accurately identifying the optimal decomposition levels to achieve the best denoising performance has become a key focus in both academia and engineering practice [5,6].

The current methods for determining the optimal wavelet denoising decomposition levels can be classified into two categories as follows: (1) methods based on noise characteristics and (2) methods based on signal characteristics [7]. Methods based on noise characteristics typically assume that the noise in the signal is white noise, which determine the decomposition level by setting hypothesis test conditions. If these conditions are exceeded, then the decomposition level is rejected. Common tests include the Jarque–Bera test and the Kolmogorov–Smirnov test [8]. By assessing whether the wavelet coefficients at each level exhibit white noise characteristics, a reasonable decomposition level can be adaptively selected. However, relying solely on noise characteristics for qualitative judgment is often inaccurate because multiple decomposition levels may exist, making it challenging to identify a single optimal level [8,9,10].

Another category of methods relies on the inherent characteristics of the signal, utilizing indicators such as the signal-to-noise ratio (*SNR*), root mean square error (*RMSE*), smoothness (*r*) and correlation coefficient (*R*) to guide the selection of decomposition levels [5]. However, the unknown true value of the signal makes it difficult for a single evaluation indicator to assess the denoising effect accurately, resulting in significant limitations. Numerous scholars have proposed weighting methods, such as the entropy weight method and the coefficient of variation method, which extend and integrate traditional metrics, such as *SNR*, *RMSE* and smoothness, to quantitatively identify the inflection points of metric changes, thereby determining the optimal decomposition level for wavelet denoising more precisely [11,12]. Nonetheless, the methods for determining optimal decomposition levels based on signal characteristics continue to face challenges, including indicator selection, the choice of weighting methods and the evaluation of noise statistical characteristics [13].

Overall, while the two aforementioned methods have achieved notable success, they still exhibit limitations in accuracy and the interpretation of physical significance. Furthermore, studies on the modeling and analysis of Gaussian white noise remain relatively limited. Therefore, this study integrates the statistical characteristics of noise with the mathematical properties of signals by employing the Jarque–Bera test for qualitative noise analysis. By integrating this approach with a weighted combination method, the optimal wavelet decomposition level is determined, leading to more effective wavelet denoising. A theoretical analysis and case studies demonstrate that the proposed method exhibits clear geometric and physical significance. The algorithm is straightforward, highly accurate and practical for determining the optimal decomposition level, offering a broad applicability in engineering practice.

## 2. Wavelet De-Noising Function Model

For a square-integrable function ψ(t) in the real number space $L^{2}\left(R\right)$, its Fourier transform satisfies the following equation [14,15]:

(1) $$\int_{R}\frac{{\left|\hat{\psi }(t)\right|}^{2}}{\left|t\right|}dt<\infty$$

where $\hat{\psi }(t)$ represents the Fourier transform of ψ(t), which is designated as the wavelet function. The collection of functions generated by the wavelet function ψ(t) through the scale factor *a* and the translation factor *b* is referred to as the continuous wavelet, specifically,

(2) $$\psi_{a,b}(t)=\frac{1}{\sqrt{a}}\psi \left(\frac{t-b}{a}\right)$$

where *a* > 0 and *b* are the real numbers.

The continuous wavelet transform of the one-dimensional noisy signal *S* is given as follows:

(3) $$W_{S}\left(a,b\right)=\langle S,\psi_{a,b}\rangle ={\left|a\right|}^{-1/2}\int S\left(t\right)\bar{\psi }\left(\left(t-b\right)/a\right)dt$$

where $\bar{\psi }$ denotes the conjugate of ψ. In practical engineering applications, continuous wavelets are commonly discretized by selecting discrete values for the scale factor *a* and the translation factor *b*, thereby facilitating analysis and processing using computers. This process is defined as follows:

(4) $$a=a_{0}^{j},b=ka_{0}^{j}b_{0}\;j,k\in Z$$

where *j* and *k* represent the scale factor and translation factor of the discrete wavelet, respectively.

The discrete wavelets are defined as follows:

(5) $$D_{S}(j,k)=\langle S,\psi_{j,k}\rangle ={\left|a_{0}\right|}^{-j/2}\int_{-\infty }^{+\infty }S\left(t\right)\bar{\psi }\left({a_{0}}^{-j}t-kb_{0}\right)dt$$

Binary wavelets are especially well suited for computer analysis and provide a high computational efficiency. The scale factor is usually discretized using a binary approach, while the translation factor is discretized in multiples of binary integers. In the formula for the discrete wavelet basis function, *a*_0_ is set to 2 and *b*_0_ is set to 1, with the variation defined as follows:

(6) $$d_{j,k}=2^{-j/2}\int_{-\infty }^{+\infty }S\left(t\right)\bar{\psi }\left(2^{-j}t-k\right)dt$$

Additionally, based on the conversion formulas for exponents and logarithms,

(7) $$N=2^{J}\Leftrightarrow J=\log_{2}\left(N\right)$$

where *J* represents the maximum level of wavelet decomposition, while *N* denotes the length of the noisy signal *S*. The maximum decomposition level depends solely on the length *N* of the signal. In this paper, the maximum decomposition level is determined by taking the floor of *J* = log_2_(*N*).

**Figure 1:**
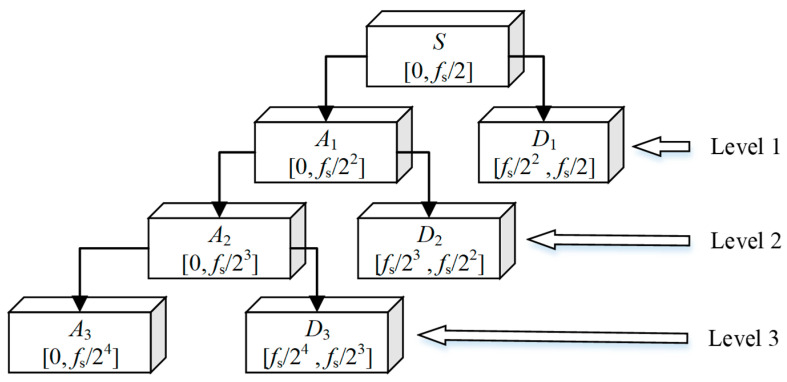
Diagram of the discrete wavelet decomposition transform.

In practical engineering applications, effective signals are generally characterized by low-frequency components or relatively stable states, while noise is predominantly concentrated in the high-frequency range. Therefore, this paper employs a multi-level wavelet decomposition method to process noise. The rules governing the discrete wavelet transform (DWT) decomposition are illustrated in Figure 1.

Figure 1 illustrates the discrete wavelet decomposition transform, where *f*_s_ denotes the sampling frequency, *A*_i_ represents the low-frequency signal component in the wavelet decomposition and *D_i_* signifies the high-frequency signal component. Noise is typically incorporated into *D_i_*, with the subscript *i* indicating the corresponding decomposition level. Subsequently, thresholding is applied to the wavelet coefficients, and the resulting signal is reconstructed to achieve the goal of denoising.

## 3. Qualitative Analysis of Gaussian White Noise

Common methods for assessing normality include the skewness–kurtosis test and the Jarque–Bera test. Relevant studies indicate that, across varying sample sizes, the skewness and kurtosis test statistics fail to control the probability of a Type I error at the significance level of 0.05; thus, their use for normality assessment is not advised.

However, when the sample size exceeds 50, the Jarque–Bera test effectively controls the probability of a Type I error at the significance level. Furthermore, while controlling the probability of z Type I error, the probability of a Type II error for the Jarque–Bera test is significantly lower than that of other test statistics. Moreover, when the sample size exceeds 100, the probability of committing a Type II error using the Jarque–Bera test approaches zero [1,16,17,18].

Consequently, this paper employs the Jarque–Bera test to conduct a qualitative analysis of Gaussian white noise, thereby ensuring its validity.

The skewness test examines the symmetry of the data distribution, and its mathematical expression is as follows [19]:

(8) $$Skewness=\frac{\frac{1}{N}\sum_{i=1}^{N}{\left(S\left(t_{i}\right)-E\left(S\right)\right)}^{3}}{{\left(\sqrt{\frac{1}{N}\sum_{i=1}^{N}{\left(S\left(t_{i}\right)-E\left(S\right)\right)}^{2}}\right)}^{3}}$$

The skewness of a normal distribution is 0. If the sample skewness is close to zero, this indicates that the sample data are symmetrically distributed around the mean. A negative skewness indicates that there are more data points below the mean than above, whereas a positive skewness suggests the opposite.

The kurtosis test examines the sharpness of the data distribution and the lengths of the two tails, and its mathematical expression is as follows:

(9) $$Kurtosis=\frac{\frac{1}{N}\sum_{i=1}^{N}{\left(S\left(t_{i}\right)-E\left(S\right)\right)}^{4}}{{\left(\sqrt{\frac{1}{N}\sum_{i=1}^{N}{\left(S\left(t_{i}\right)-E\left(S\right)\right)}^{2}}\right)}^{4}}-3$$

The kurtosis is influenced by a few extreme values. The larger the kurtosis, the more extreme the deviation of the values from the mean. The kurtosis of the normal distribution is 0. If the sample kurtosis is close to 0, it indicates that the sample data are relatively flat without prominent peaks or steep features.

When the sample size N→∞, the sample skewness and kurtosis tend to be a normal distribution:

(10) $$\left\{\begin{array}{l} E\left(Skewness\right)\to 0\;E\left(Kurtosis\right)\to 0\\ D\left(Skewness\right)\to \frac{6}{N}\;D\left(Kurtosis\right)\to \frac{24}{N} \end{array}\right.$$

where $E\left(\cdot \right)$ represents the mathematical expectation and $D\left(\cdot \right)$ represents the variance.

The null hypothesis *H*_0_: the population follows a normal distribution, and the test statistic is as follows:

(11) $$JB=\frac{N}{6}(Skewness^{2}+\frac{1}{4}Kurtosis^{2})$$

where *JB* represents the chi-square test statistic, and its value is less than the value $\chi_{P}^{2}$, indicating that the quantiles for which Equation (12) is valid are presented in Table 1.

(12) $$P\left\{JB\leq \chi_{P}^{2}\right\}=\int_{0}^{\chi_{P}^{2}}K_{2}\left(x\right)dx$$

where *K*_2_ (*x*) denotes the probability density function of the chi-square distribution with 2 degrees of freedom.

From Table 1, *v* denotes the degrees of freedom, which is set to 2 in this study. *P* indicates the confidence level, with 95% selected as the standard for evaluating normality. The value of $\chi_{P}^{2}$ is determined by the corresponding values of *P* and *v*.

In practical applications, the selection of the confidence level should take into account factors such as the problem’s context, data reliability, and sample size.

## 4. Quantitative Evaluation of the Optimal Decomposition Level of Wavelets

The optimal decomposition level for wavelet denoising, based on signal characteristics, is determined by evaluating the statistical properties of the denoised signal. Common evaluation indicators include the signal-to-noise ratio (*SNR*), Root Mean Square Error (*RMSE*), correlation coefficients (*R*), and smoothness (*r*), each representing distinct physical meanings and characteristics. If a pair of metrics, representing different perspectives (such as signal detail and overall characteristics), are negatively correlated, and both values improve together (whether larger or smaller), then theoretically, a composite metric can quantify the optimal wavelet decomposition level. This serves as the theoretical foundation for the composite metric evaluation methods.

### 4.1. Selection of Assessment Indicators

The key to selecting integrated metrics lies in identifying methods that quantitatively describe denoised signal characteristics from various perspectives, such as signal details and approximation information. The common metrics used to evaluate wavelet denoising performance include *SNR*, *RMSE*, *R* and *r*, which are defined as follows [20]:

(13) $$RMSE=\sqrt{\frac{1}{N}\sum_{i=1}^{N}{\left(S\left(t_{i}\right)-\hat{S}\left(t_{i}\right)\right)}^{2}}$$

(14) $$SNR=10\mathrm{lg}\frac{\sum_{i=1}^{N}{\left|S\left(t_{i}\right)\right|}^{2}}{\sum_{i=1}^{N}{\left|S\left(t_{i}\right)-\hat{S}\left(t_{i}\right)\right|}^{2}}$$

(15) $$R=\frac{\sum_{i}^{N}S\left(t_{i}\right)\hat{S}\left(t_{i}\right)-\sum_{i}^{N}S\left(t_{i}\right)\sum_{i}^{N}\hat{S}\left(t_{i}\right)}{\sqrt{\sum_{i}^{N}S{\left(t_{i}\right)}^{2}-{\left(\sum_{i}^{N}S\left(t_{i}\right)\right)}^{2}}\times \sqrt{\sum_{i}^{N}\hat{S}{\left(t_{i}\right)}^{2}-{\left(\sum_{i}^{N}\hat{S}\left(t_{i}\right)\right)}^{2}}}$$

(16) $$r=\frac{\sum_{i=1}^{N-1}{\left[\hat{S}\left(t_{i+1}\right)-\hat{S}\left(t_{i}\right)\right]}^{2}}{\sum_{i=1}^{N-1}{\left[S\left(t_{i+1}\right)-S\left(t_{i}\right)\right]}^{2}}$$

where *S* represents the original signal sequence and $\hat{S}$ represents the denoised signal sequence. When the true value is known, *S* refers to the true signal sequence without noise; when the true value is unknown, *S* refers to the noisy observed signal sequence.

The *RMSE* reflects the overall bias of the signal, with smaller values indicating superior denoising performance. The *SNR* describes the impact of noise on the overall signal, with a higher *SNR* generally associated with improved filtering efficacy. The smoothness indicates the local variation within the signal, particularly highlighting the presence of abrupt changes; a smoother signal correlates with a lower smoothness index value, signifying enhanced denoising effectiveness. The correlation coefficient quantifies the degree to which the denoised signal aligns with the theoretical reference signal, with values approaching 1 indicating a closer resemblance and a better fit.

To further illustrate the limitations of employing a single metric when the original clean signal is unknown, this paper uses the determination of the optimal decomposition level based on a single metric as a case study, employing the wavelet threshold denoising method to evaluate the denoising effects of the simulated signal across various decomposition levels. To ensure that the simulated signal closely resembles the actual monitored signal, three distinct frequency sine signals, linear signals and noise signals are selected and superimposed for analysis, with the corresponding expressions provided as follows:

(17) $$S=2.5\sin\left(2\pi t/500\right)+1.5\sin(2\pi t/300)+\sin\left(2\pi t/60\right)+0.003t+\varepsilon$$

where *t* represents the time series, while ε represents the simulated noise sequence.

**Figure 2:**
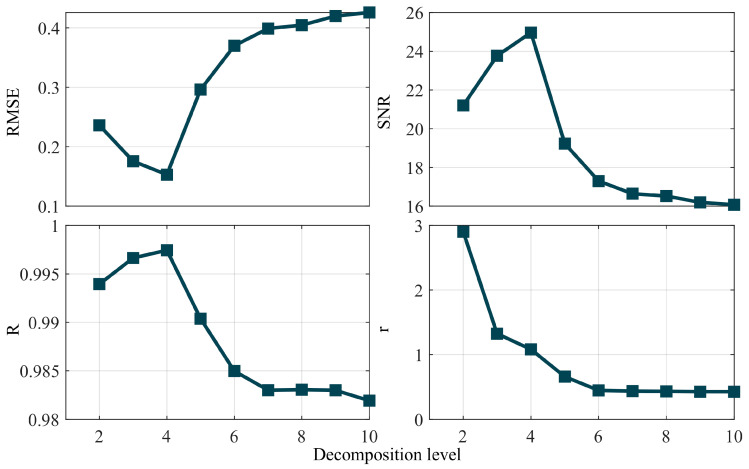
Trend of a single evaluation indicator for a simulated signal with a known true value.

**Figure 3:**
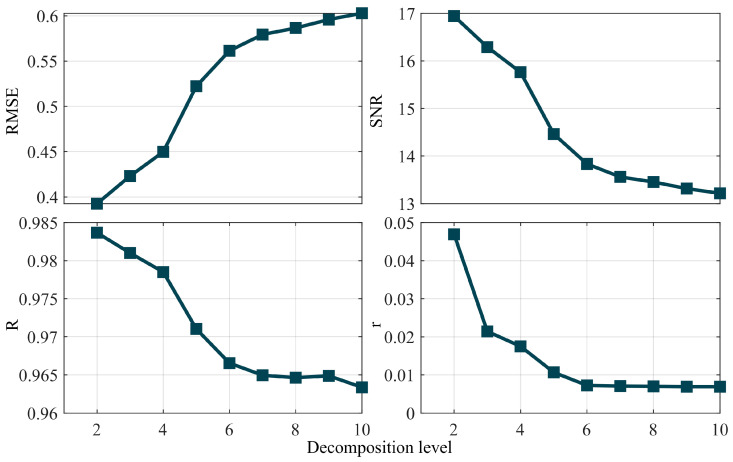
Trend of a single evaluation indicator for a simulated signal with an unknown true value.

The *SNR* of the simulated data is 15 dB, characterized by a signal length of 1024 sampling points and a sampling frequency of 1 Hz. Denoising is performed using the db4 wavelet basis function, with the decomposition levels ranging from 2 to 10; the optimal decomposition level is determined based on the four aforementioned traditional metrics. The resulting data are presented in the form of a trend. Figure 2 and Figure 3 illustrate the trend lines obtained under the conditions of known and unknown true values, respectively.

Figure 2 and Figure 3 illustrate the trend lines derived under conditions of known and unknown true values, respectively. An analysis of Figure 2 reveals that, under conditions of known true values, the optimal decomposition level determined by the *RMSE*, *SNR* and correlation coefficient is 4, whereas the smoothness metric does not yield a conclusive judgment. An analysis of Figure 3 indicates that, under the conditions of unknown true values, none of the four metrics can accurately determine the optimal decomposition level. Additionally, the *SNR*, correlation coefficient and smoothness decrease as the decomposition level increases, whereas the *RMSE* increases with rising decomposition levels. Therefore, under conditions of unknown true values, no traditional single metric suffices to determine the optimal wavelet decomposition level.

In practical applications, the reliability of the correlation coefficient is limited due to the absence of known true values for the signals. Consequently, this paper employs the previously mentioned simulated data to develop a composite evaluation indicator that incorporates geometric significance, physical significance and the Pearson correlation coefficient among the three metrics: *RMSE*, *SNR*, and smoothness.

Given that the base ranges and units of the three metrics differ, these metrics are normalized to a common range of [0, 1] for easier comparison. Furthermore, considering that smaller values are preferable for RMSE and smoothness, whereas larger values are advantageous for *SNR*, it is essential to apply a trend adjustment to the *SNR*. The specific processing formulas are presented as follows [21]:

(18) $$PRMSE_{i}=\frac{RMSE_{i}-\min(RMSE)}{\max(RMSE)-\min(RMSE)}$$

(19) $$PSNR_{i}=\frac{\max(SNR)-SNR_{i}}{\max(SNR)-\min(SNR)}$$

(20) $$Pr_{i}=\frac{r_{i}-\min(r)}{\max(r)-\min(r)}$$

where *PRMSE*, *PSNR* and *Pr* represent the normalized and trend-adjusted RMSE, *SNR* and smoothness, respectively, with the subscript *i* indicating the wavelet decomposition level, where *i* = 1, 2, …, 10.

A Pearson correlation analysis was performed on the three metrics following standardization and trend adjustment. The correlations among the metrics are presented in Table 2.

As indicated in Table 2, a significant correlation exists between the *SNR* and *RMSE*, with a Pearson correlation coefficient of 0.996, indicating that both metrics characterize the detailed information of the signal. Consequently, this study discards *SNR* and selects *RMSE* as the composite evaluation indicator. Simultaneously, to better capture the overall characteristics of the denoised signal, smoothness is chosen as an additional composite evaluation indicator. *RMSE* and smoothness measure the detailed information and overall approximation of the signal, respectively. Furthermore, these two metrics are negatively correlated, with a correlation coefficient of −0.796.

When employing a composite evaluation indicator that integrates *RMSE* and smoothness, as the decomposition level increases, the evaluation indicator will inevitably attain an extremum. The physical significance of this extremum is that it represents the optimal balance between preserving detailed information and ensuring the overall approximation of the signal, at which point the decomposition level is deemed optimal.

### 4.2. Construction of Composite Assessment Indicators

Due to the different ways in which *RMSE* and smoothness describe signal features, their weights vary during the composite process, necessitating a weighting approach. The commonly used methods for assigning weights include the entropy weight method and the coefficient of variation method. The entropy weight method allocates weights based on information entropy, making it suitable for scenarios with relatively stable data and minimal subjective judgment. It is particularly advantageous when dealing with complex systems that involve large volumes of information. In contrast, the coefficient of variation method assigns weights by evaluating the data’s volatility, making it more suitable for situations with significant fluctuations or variations in the data. This method better reflects the variability and influence of the indicators. In this paper, the coefficient of variation method is used to assign different combination coefficients to both the entropy weight method and the coefficient of variation method. The resulting composite evaluation index enhances the scientific and rational allocation of weights.

#### 4.2.1. Entropy Weight Method

The entropy weight method is an objective weighting technique that establishes weights according to the information content of each indicator. A smaller entropy value for an indicator signifies a greater degree of variation and a higher richness of information provided, thereby enhancing its significance in comprehensive evaluation, resulting in a correspondingly larger weight. This study calculates the corresponding weights for the two indicators, *RMSE* and smoothness, and the specific steps are outlined as follows [12,22]:

First, calculate the contributions of the standardized and detrended *RMSE* and smoothness (r) indicators to the overall *RMSE* and smoothness across all decomposition levels, specifically at the *i*-th decomposition level.

(21) $$\begin{array}{l} P_{i}^{PRMSE}=PRMSE_{i}/\sum_{j=1}^{k}PRMSE_{j}\\ P_{i}^{Pr}=Pr_{i}/\sum_{j=1}^{k}Pr_{j} \end{array}$$

where *i* represents the decomposition level, ranging from 1 to *k*. Here, *k* denotes the highest decomposition level, which is determined by Equation (7).

Calculate the information entropy of the two main indicators.

(22) $$\begin{array}{l} e_{PRMSE}=-\frac{1}{\mathrm{In}k}\sum_{i=1}^{k}P_{i}^{PRMSE}lnP_{i}^{PRMSE}\\ e_{Pr}=-\frac{1}{\mathrm{In}k}\sum_{i=1}^{k}P_{i}^{Pr}lnP_{i}^{Pr} \end{array}$$

Calculate the weights of the two evaluation indicators.

(23) $$\begin{array}{l} w_{PRMSE}=\frac{1-e_{PRMSE}}{\left(1-e_{PRMSE}\right)+\left(1-e_{Pr}\right)}\\ w_{Pr}=\frac{1-e_{Pr}}{\left(1-e_{PRMSE}\right)+\left(1-e_{Pr}\right)} \end{array}$$

Calculate the composite evaluation indicator.

(24) $$F=w_{PRMSE}\cdot PRMSE+w_{Pr}\cdot Pr$$

By analyzing the definitions of *RMSE* and smoothness, as well as their weight allocation process, it can be concluded that a smaller composite evaluation indicator corresponds to a more effective denoising effect at the decomposition level, leading to a more thorough removal of noise. Therefore, the decomposition level corresponding to the minimum value of the composite evaluation indicator is considered to be the theoretical optimal level based on the entropy weight method.

#### 4.2.2. Variation Coefficient Method

The coefficient of variation method is an objective statistical weighting technique employed to assess the degree of variation for each indicator. In the evaluation system, indicators with greater variation are more effective in highlighting the differences between evaluation units, and thus should be assigned higher weights, while indicators with smaller variation should receive lower weights. This method determines the significance of each indicator based on its statistical characteristics [11,21].

First, calculate the coefficient of variation based on the mean and standard deviation.

(25) $$\begin{array}{l} CV_{PRMSE}=\frac{\sigma_{PRMSE}}{\mu_{PRMSE}}\\ CV_{Pr}=\frac{\sigma_{Pr}}{\mu_{Pr}} \end{array}$$

The weight of the indicator is determined by the coefficient of variation.

(26) $$\begin{array}{l} W_{PRMSE}=\frac{CV_{PRMSE}}{CV_{PRMSE}+CV_{Pr}}\\ W_{Pr}=\frac{{\mathrm{CV}}_{Pr}}{{\mathrm{CV}}_{PRMSE}+{\mathrm{CV}}_{Pr}} \end{array}$$

where $\sigma_{PRMSE}$ and $\mu_{PRMSE}$ represent the standard deviation and mean of *RMSE*, respectively; $\sigma_{Pr}$ and $\mu_{Pr}$ represent the standard deviation and mean of smoothness; $CV_{PRMSE}$ and $CV_{Pr}$ represent the coefficient of variation for *RMSE* and smoothness; and $W_{PRMSE}$ and $W_{Pr}$ represent the weights of *RMSE* and smoothness, respectively.

Calculate the composite evaluation indicator.

(27) $$T=W_{PRMSE}\cdot PRMSE+W_{Pr}\cdot Pr$$

Similarly, a smaller composite evaluation indicator corresponds to a more effective denoising effect at the decomposition level, indicating a more thorough removal of noise. Therefore, the decomposition level corresponding to the minimum value of the composite evaluation indicator is considered to be the theoretical optimal level based on the coefficient of variation method.

#### 4.2.3. Combined Weighting Method

After determining the weights of the two aforementioned weighting methods, to address their respective limitations and minimize the loss of valuable information, a combined weighting method is developed by integrating the entropy weight method with the coefficient of variation method. This ensures that the indicator weights are more objective and justifiable, ultimately leading to the final comprehensive weight. Let the weight vector determined by the coefficient of variation method be represented as *T*, and the weight vector determined by the entropy weight method be represented as *F*. By applying an addition-based ensemble method, the final comprehensive weight vector *H* is derived.

(28) H=αT+βF

where α and β represent the undetermined coefficients of the combined weighting method, which can be determined by applying the difference coefficient method.

(29) $$\alpha =\frac{R_{Em}\cdot m}{m-1}\;\beta =1-\alpha$$

(30) $$R_{Em}=\frac{2}{m}(\begin{array}{c} 1\cdot p_{1}+2\cdot p_{2}+\cdot \cdot \cdot +m\cdot p_{m} \end{array})-\frac{m+1}{m}$$

where *T* and *F* represent the weights of the coefficient of variation method and the entropy weight method, respectively; $R_{Em}$ denotes the coefficient of variation; and *p*_1_, *p*_2_, …, *p*_m_ represent the weights of the coefficients of variation for each indicator, arranged in an ascending order. *m* denotes the number of combinations for weighting combinations, and in this study, *m* is set to 2, allowing Equation (30) to be further derived as follows:

(31) $$R_{Em}=(\begin{array}{c} 1\cdot \min\left\{W_{PRMSE},W_{Pr}\right\}+2\cdot \max\left\{W_{PRMSE},W_{Pr}\right\} \end{array})-1.5$$

**Figure 4:**
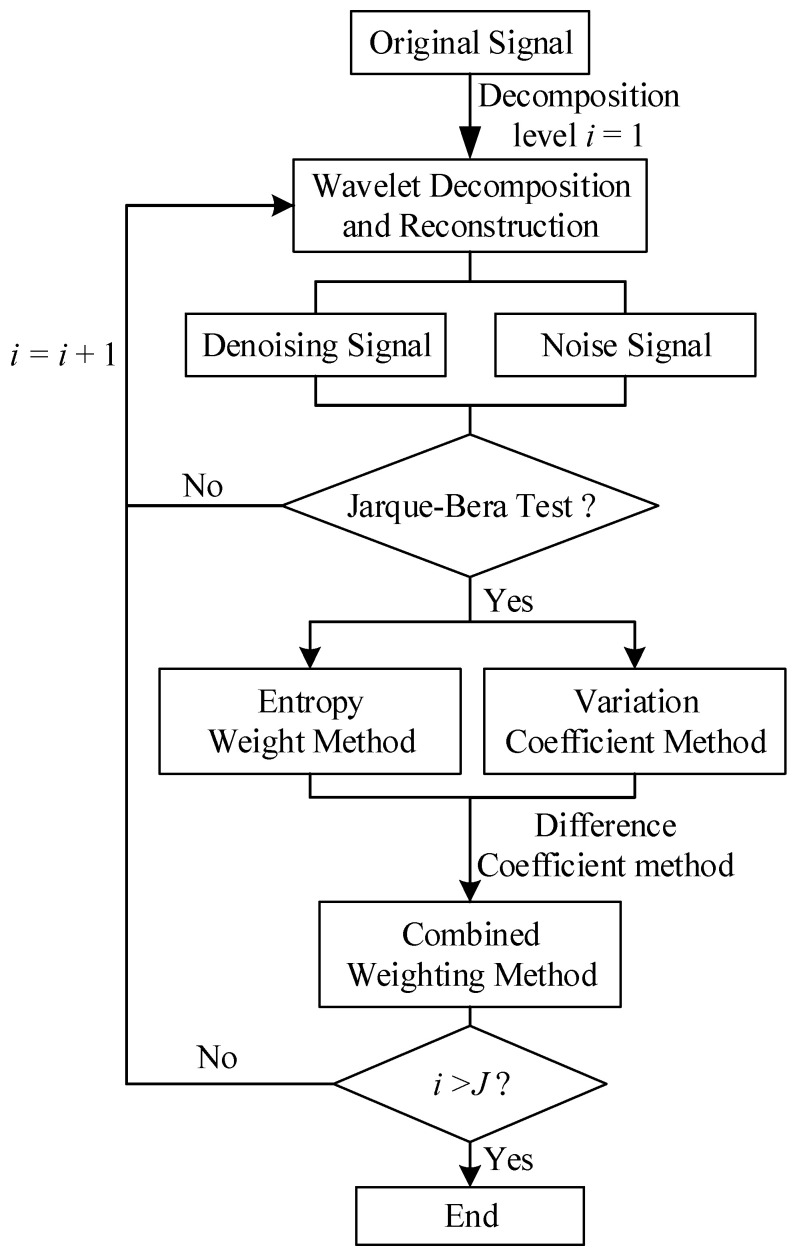
The flowchart for calculating the optimal decomposition levels.

The equation presented above functions as the weighting formula for the combined weighting method. The computational process for determining the optimal decomposition levels, as proposed in this paper, is illustrated in Figure 4.

The design concept of this paper is illustrated in Figure 4. The original noisy signal is decomposed into noise and denoised components using a wavelet thresholding function. First, in the noise signal extraction phase, the Jarque–Bera test is applied to ensure that the extracted noise adheres to the characteristics of Gaussian white noise, thereby preventing inadequate or distorted denoising. The information from decomposition levels that pass the Jarque–Bera test is retained, while levels that do not pass are discarded.

Next, in the denoised signal evaluation phase, the denoising performance is assessed using two metrics: *RMSE* and smoothness. Since these metrics describe different aspects of the signal, a weighted composite indicator is used for evaluation. To address the limitations of relying on a single weighting method, a combined weighting strategy is proposed, integrating the entropy method and the coefficient of variation method. The combined weighting coefficient is then calculated using the difference coefficient method.

By combining the evaluation strategies for noise signal extraction and denoised signal assessment, the optimal decomposition level for the noisy signal at specific wavelet coefficients is determined by the extremum of the composite indicator, traversing from the first decomposition level to the highest. This represents the design concept of this paper.

## 5. Simulation Experiments and Practical Engineering Applications

### 5.1. Simulation Experiment

To accurately compare and validate the effectiveness of the composite evaluation indicator introduced in this study in determining the optimal decomposition level, the simulation signals with known true values derived from Equation (17) are employed for a wavelet denoising analysis.

The control variable method is employed to process noisy signals at varying decomposition levels while maintaining the simulated noise constant. Concurrently, the simulated noise with varying SNRs is analyzed while holding all other parameters constant. Specifically, the db4 wavelet basis function, the ‘sqtwolog’ threshold criterion and the soft-thresholding processing function are utilized. To validate the applicability of this approach, the methods proposed by Wang (index *F*) and Zhu (index *T*) are employed as comparison benchmarks [11,12].

The simulated signals with noise levels of 9 dB and 15 dB are used as examples. The *RMSE* and *SNR* of the signals are initially calculated using known true values, where the original signals are free of noise. Subsequently, the *RMSE* and smoothness are evaluated when the true values are unknown, and the original signals include noise. The study also computes the proposed composite evaluation indicator in conjunction with the relevant indicators from Wang’s (index *F*) and Zhu’s (index *T*) methods. The detailed results are provided in Table 3 and Table 4.

Table 3 shows that when denoising is performed using the db4 wavelet basis, the *RMSE* achieves its minimum and the *SNR* reaches its maximum at a decomposition level of 4 under known true values, indicating that level 4 is optimal. When the true values are unknown, increasing the decomposition level results in a continuous increase in *RMSE* and a decline in smoothness *r*, which is consistent with a prior analysis, which makes determining the optimal level challenging. Based on the composite evaluation indicator, the minimum value is also observed at level 4, confirming it as the optimal decomposition level, which aligns with the actual conditions. This conclusion is supported by Wang’s method. However, Zhu’s method identifies the minimum value at level 3, suggesting an optimal decomposition level of 3, which contradicts the actual conditions.

Table 4 shows that when using the db4 wavelet basis for denoising, the optimal decomposition level is 4 under known true values. Likewise, when the true values are unknown, the composite evaluation indicator identifies level 4 as optimal, aligning with the actual conditions. However, Wang’s method identifies level 5 as optimal, while Zhu’s method identifies level 3, both of which deviate from the actual conditions. This underscores the superior accuracy and reliability of the composite evaluation indicator compared to the existing methods.

To comprehensively analyze the reliability of the proposed method and avoid misleading results due to a single wavelet basis function, a denoising analysis is conducted using Gaussian white noise with varying SNRs under unknown true values, aiming to derive a composite evaluation indicator for determining the optimal decomposition level. The optimal decomposition level determined under known true values serves as a reference for comparing the proposed method with the existing methods, as detailed in Table 5.

To comprehensively assess the reliability of the method proposed in this paper and to avoid potential bias from a single data set, a control variable approach is employed. With the wavelet basis function held constant, Gaussian white noise at varying SNRs is introduced for denoising analysis, and the optimal decomposition level for each *SNR* condition is determined. The optimal decomposition level obtained under known ground truth is used as a reference, and the proposed method is compared with the existing methods, as detailed in Table 5. Additionally, under constant *SNR* conditions, different wavelet basis functions are selected for the denoising analysis to evaluate the optimal decomposition level for each wavelet. The method for determining the optimal decomposition level proposed in this paper is then compared with the existing methods to assess its performance, with the data processing results presented in Table 6.

The analysis of Table 5 and Table 6 reveals that the accuracy of determining the optimal decomposition level varies across different algorithms. Under the same wavelet basis function, the optimal decomposition level varies across different *SNR* conditions. The proposed method achieves a 100% accuracy, while Wang’s method and Zhu’s method have accuracies of 83% and 50%, respectively. Under the same *SNR* conditions, different wavelet basis functions also yield varying optimal decomposition levels. The proposed method consistently maintains a 100% accuracy, whereas Wang’s method and Zhu’s method achieve accuracies of 67% and 33%, respectively.

This demonstrates that the method presented in this paper outperforms the existing approaches in terms of accuracy and reliability. It can be widely applied to signals with different wavelet basis functions and varying *SNR* conditions, making it a robust and reliable method for determining the optimal decomposition level for wavelet denoising.

### 5.2. Engineering Practical Applications

#### 5.2.1. Function Model

According to Kepler’s third law, the square of a satellite’s orbital period is proportional to the cube of the semi-major axis of its elliptical orbit. Using the parameters *a_s_* and *d_n_* from the broadcast ephemeris file, this paper derives the following formula for the mean angular velocity *n*:

(32) $$n=\sqrt{GM}a_{s}^{-3}+d_{n}=\frac{2\pi }{T}$$

where *a_s_* denotes the square root of the semi-major axis of the satellite’s orbital ellipse, *d_n_* represents the correction factor for the mean angular velocity, *G* is the gravitational constant and *M* is the Earth’s mass, while *T* represents the rotational period of the spacecraft [23].

Based on the calculation from Equation (32), the orbital period of geostationary Earth orbit (GEO) satellites is approximately 23 h and 56 min. Therefore, this study assumes 23 h and 56 min as a single period to calculate the pseudorange multipath of GEO satellites and applies wavelet denoising.

The optimal wavelet decomposition level is determined by analyzing noise signals in both the time and frequency domains at various decomposition levels. Furthermore, multipath periodic modeling experiments use the wavelet-denoised multipath time series to correct the undenoised multipath time series of the subsequent cycle. Finally, the *RMSE* of the corrected results is calculated to verify the optimal wavelet decomposition level.

The pseudorange multipath (MP) combination is achieved by combining the pseudorange observation *P* at frequency *i* with the carrier phase observations *φ* at frequencies *i* and *j*, thereby effectively reducing the impact of tropospheric and ionospheric delays. The corresponding expression is: [24]:

(33) $$MP_{i}=P_{i}-\frac{f_{i}^{2}+f_{j}^{2}}{f_{i}^{2}-f_{j}^{2}}\lambda_{i}\varphi_{i}+\frac{2f_{j}^{2}}{f_{i}^{2}-f_{j}^{2}}\lambda_{j}\varphi_{j}-B_{i}$$

where *λ* is the wavelength of the carrier phase, corresponding to the frequency *f*, and the bias term *B_i_* is expressed as:

(34) $$B_{i}=\frac{f_{i}^{2}+f_{j}^{2}}{f_{i}^{2}-f_{j}^{2}}\lambda_{i}N_{i}+\frac{2f_{j}^{2}}{f_{i}^{2}-f_{j}^{2}}\lambda_{j}N_{j}+\Psi$$

where *N* represents the integer ambiguity, and Ψ denotes the time-invariant component of hardware delays and multipath effects.

#### 5.2.2. Analysis of Experiments

**Figure 5:**
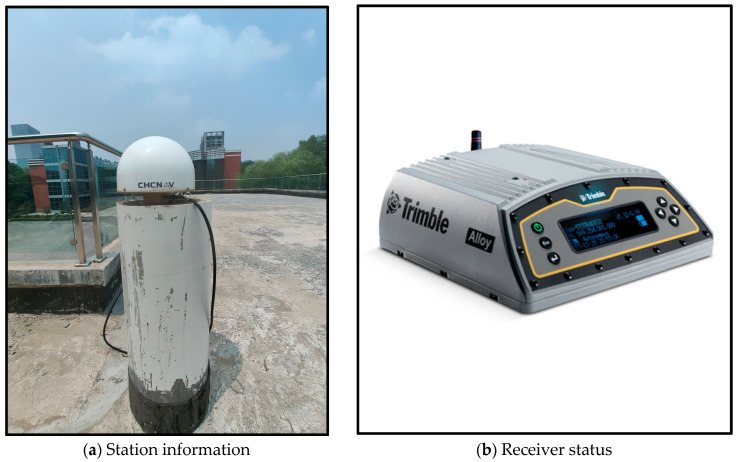
Basic information of the station.

This study applies the proposed method to model the GNSS pseudorange multipath effects, utilizing data sourced from measurements taken at a fixed station within a laboratory building in Zhengzhou during DOY (Day of year) 151–152 of 2024, with a sampling interval of 30 s collected using a TRIMBLE ALLOY receiver. The detailed information regarding the station and receiver is provided in Figure 5.

**Figure 6:**
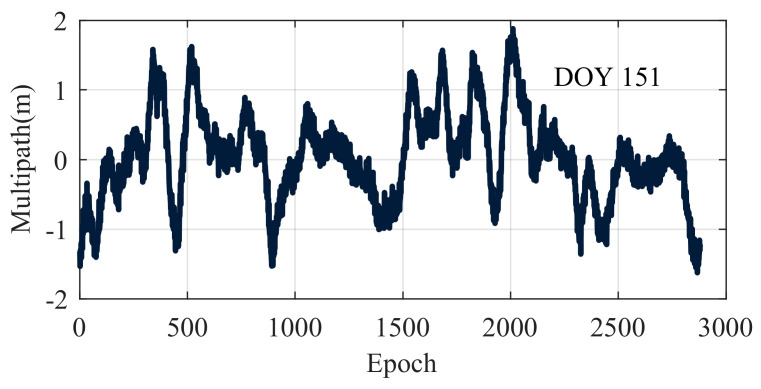
Real observation data.

**Figure 7:**
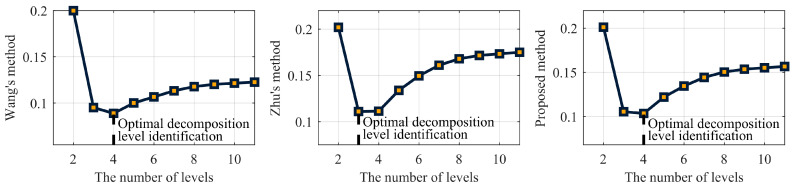
Determining the optimal decomposition level for pseudorange multipath signals.

Before applying wavelet denoising, it is essential to maintain the consistency of other parameters. The ‘rigrsure’ threshold selection criterion and the soft threshold function were employed, using the db5 wavelet basis function with decomposition levels ranging from 1 to 11. The optimal decomposition level was determined by integrating Wang’s method, Zhu’s method and the proposed method using a controlled variable approach. The MP time series at the C60 satellite B1I frequency point is depicted in Figure 6, and the corresponding indicator data for the decomposition levels are presented in Table 7. Figure 7 illustrates the graph used to determine the optimal decomposition level of the pseudorange multipath signal.

**Figure 8:**
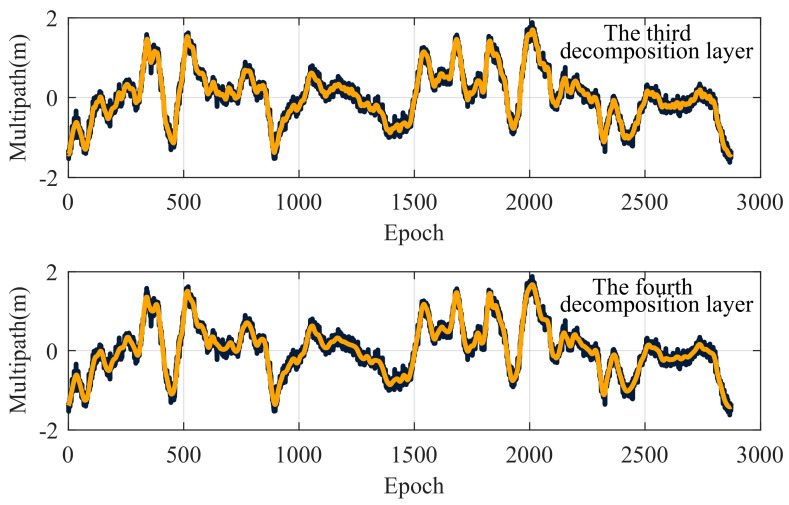
Time-domain curves of the original (black line) and denoised (orange line) signals.

**Figure 9:**
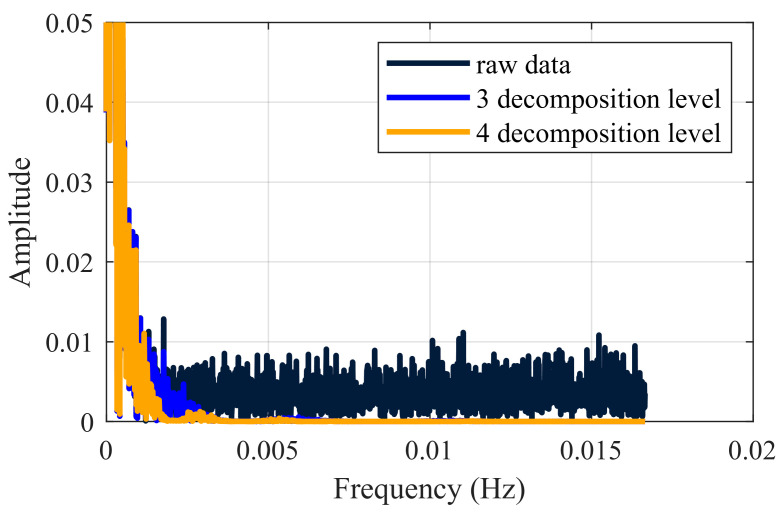
Spectrum curves of the original and denoised signals.

Table 6 and Figure 7 show that the optimal decomposition levels differed depending on the wavelet denoising method used. Wang’s method and the proposed method both identify level 4 as optimal, whereas Zhu’s method selects level 3. To visually evaluate the accuracy of these methods, the time-domain and spectral curves for the denoised signals at the third and fourth decomposition levels were plotted, as shown in Figure 8 and Figure 9, respectively.

Figure 8 demonstrates that the wavelet-denoised signal preserves the overall trend of the original signal. Compared to the 3-level decomposition, the 4-level denoised signal exhibits a smoother peak curve, a more stable waveform and more comprehensive noise reduction. Figure 9 presents the single-sided amplitude spectrum, where denoising primarily eliminates the irrelevant high-frequency details while preserving the essential low-frequency components, aligned with the principles of wavelet denoising. The 4-level decomposition removes high-frequency noise more effectively than the 3-level one, yielding a higher *SNR*. Thus, the 4-level decomposition achieves the optimal denoising effect under these conditions, indirectly confirming the effectiveness of the composite evaluation indicators.

**Figure 10:**
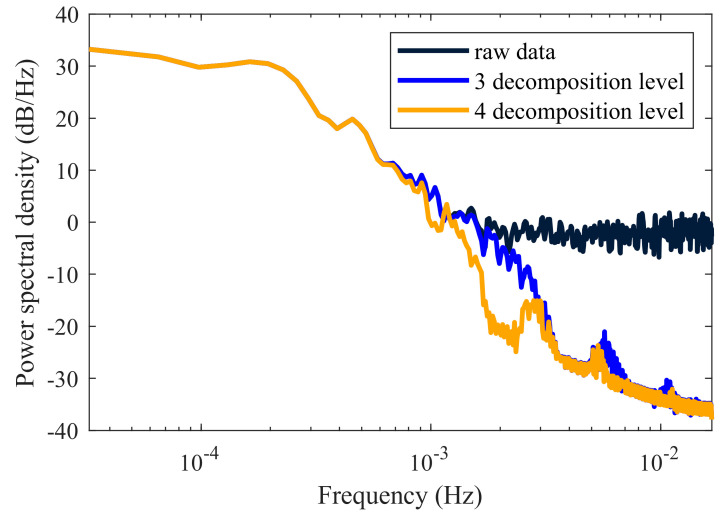
Power spectral density plot of the original and denoised signals.

To better assess the wavelet denoising effect at different decomposition levels, Figure 10 presents the power spectral density estimates of the original signal and denoised signals after the third- and fourth-level decompositions. The figure indicates that the denoised signal retains valuable low-frequency information, eliminates high-frequency noise and appropriately preserves transitional mid-frequency details. Compared to the three-level decomposition, the four-level decomposition eliminates weak noise more effectively, confirming that the four-level approach is optimal.

**Figure 11:**
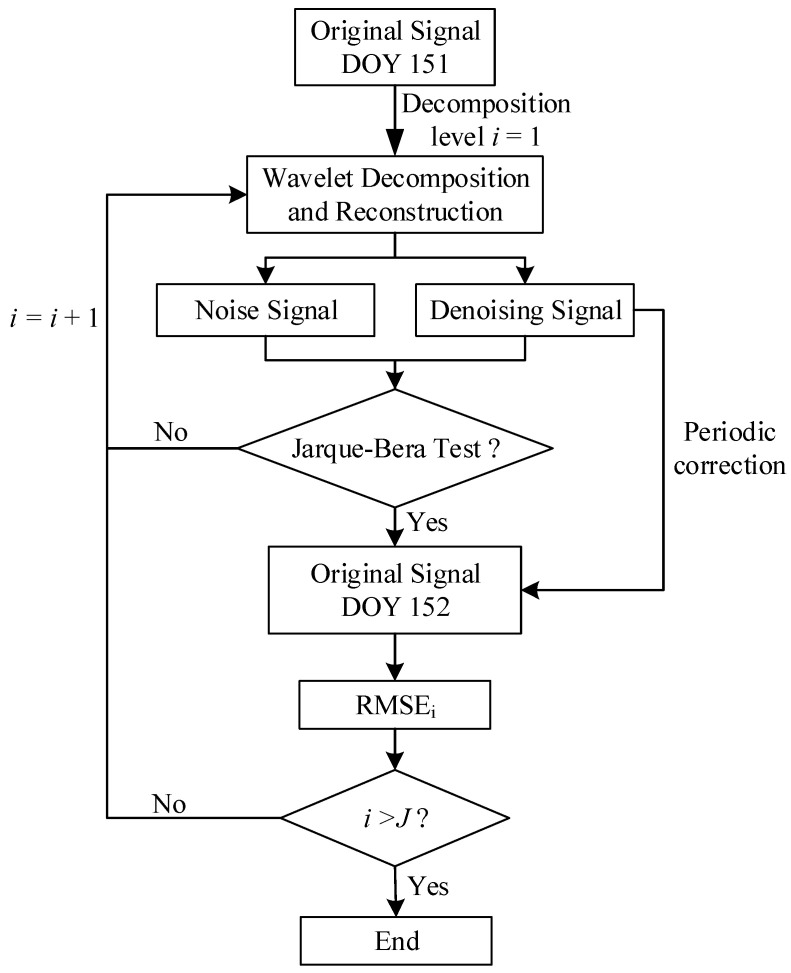
Flowchart of pseudorange multipath periodic correction.

To verify the accuracy of the optimal decomposition level determined in this paper, the orbital period of GEO satellites can be derived based on Kepler’s Third Law, with the specific calculation formula provided in Equation (32). For static reference stations, a periodic modeling analysis of a pseudorange multipath is performed. This involves denoising the pseudorange multipath data for DOY 151 using wavelet basis functions at different decomposition levels and then using the denoised signal to correct the original pseudorange multipath combination for DOY 152. The difference between the two is then calculated, and the *RMSE* value for DOY 152, after removing the periodic components, is computed. The detailed process of periodic correction is shown in Figure 11, with the statistical data presented in Table 8.

The analysis in Table 8 indicates that the *RMSE* reaches its minimum at a four-level decomposition, demonstrating an optimal performance in multipath periodic modeling. This result aligns with the conclusions from composite evaluation indicators, a time-domain analysis and a frequency-domain analysis, further validating the effectiveness of the proposed method in producing real-world data. 

**Figure 12:**
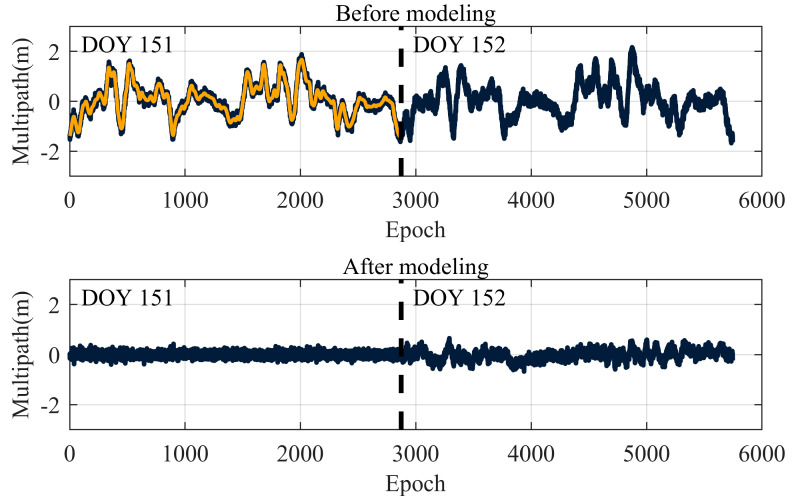
Multipath time series before and after model correction.

Figure 12 illustrates the application of the fourth-level wavelet basis function to denoise the pseudorange multipath sequence of DOY 151. The denoised signal is then used to correct the original pseudorange multipath sequence of DOY 152. The comparison of the results before and after correction is shown by the black curve, while the wavelet-denoised sequence for DOY 151 is represented by the orange curve.

Analysis of Figure 12 reveals that the optimal wavelet decomposition level determined in this study effectively removes noise from the actual data, thereby enhancing the accuracy of multipath periodic modeling.

## 6. Conclusions

This paper addresses the filtering problem of Gaussian white noise and proposes an optimal decomposition level determination method for wavelet denoising based on the combination of the Jarque–Bera test and a composite weighting approach. Through an analysis of both simulation signals and engineering examples, the proposed comprehensive evaluation metrics demonstrate superior performance and applicability. The main conclusions are as follows:

(1) In situations where the true value is unknown, a single evaluation metric cannot effectively guide the determination of the optimal decomposition level in the wavelet threshold denoising process. Therefore, a more comprehensive quality evaluation index needs to be developed.
(2) By combining the statistical characteristics of the noise and the mathematical features of the signal, an effective filtering method for Gaussian white noise is proposed to accurately determine the optimal wavelet decomposition level. First, during the noise extraction phase, the Jarque–Bera test is employed to ensure that the extracted noise conforms to the characteristics of Gaussian white noise, thus avoiding insufficient denoising or signal distortion. Next, in the signal denoising evaluation phase, *RMSE* and smoothness are selected as the evaluation metrics for denoising performance. Since these two metrics describe different aspects of the signal, a weighted composite approach is used to generate a single comprehensive evaluation index. To overcome the limitations of a single weighting method, a combination weighting strategy is introduced by integrating the entropy weighting method with the coefficient of variation method. The combination coefficient of these two methods is calculated using the difference coefficient approach, thereby yielding a new composite evaluation index. The smaller the index value, the better the denoising effect, and the more accurately the optimal decomposition level is determined.
(3) The simulation results indicate that the proposed comprehensive evaluation method can accurately determine the optimal wavelet decomposition level, whether under the same wavelet basis with varying SNRs or under the same *SNR* condition with different wavelet bases, both when true values are known and unknown. The method demonstrates high accuracy and good applicability. The effectiveness of this approach is further validated with real-world data. After applying the optimal decomposition level determined by this method for wavelet denoising, the signal’s peak domain becomes smoother, the waveform stabilizes and the denoising effect is significantly improved. Additionally, the modeling of multi-path periodicity is also enhanced.

In conclusion, the wavelet optimal decomposition level determination strategy proposed in this paper offers a higher accuracy and greater universality compared to other algorithms. It is important to note that the algorithm designed in this paper mainly targets the most common Gaussian white noise filtering problem in industrial production. However, industrial settings also involve other types of measurement noise, such as Laplacian noise or colored noise, which do not follow Gaussian distributions. These issues still require further research.

## Figures and Tables

**Table 1:** A summary table of $\chi_{P}^{2}$ percentiles corresponding to specific *P* and *v* parameters.

	*P*	0.95	0.975	0.99	0.995
*v*					
2		5.99	7.38	9.21	10.6

**Table 2:** Pearson correlation analysis statistics between indicators.

	*RMSE*	*SNR*	*r*
*RMSE*	\		
*SNR*	0.997	\	
*r*	−0.796	−0.841	\

**Table 3:** Evaluation indicators for wavelet denoising across various decomposition levels at an *SNR* of 9 dB.

Decomposition Level	Jarque–Bera Test	True Value Known		True Value Unknown		*F*	*T*	*H*
		*RMSE*	*SNR*	*RMSE*	*r*			
2	accept	0.4657	15.3004	0.8378	0.0352	0.1551	0.1683	0.1640
3	accept	0.3019	19.0651	0.9101	0.0069	0.0616	0.0927	0.0827
4	accept	0.2248	21.6263	0.9440	0.0044	0.0574	0.0937	0.0820
5	accept	0.5122	14.4730	1.0691	0.0019	0.0672	0.1207	0.1035
6	accept	0.6220	12.7870	1.1356	0.0008	0.0732	0.1357	0.1156
7	accept	0.6763	12.0590	1.1768	0.0007	0.0791	0.1471	0.1252
8	accept	0.7139	11.5898	1.1924	0.0007	0.0814	0.1514	0.1289
9	accept	0.7454	11.2140	1.2126	0.0006	0.0843	0.1570	0.1336
10	accept	0.7649	10.9897	1.2271	0.0006	0.0865	0.1611	0.1371

**Table 4:** Evaluation indicators for wavelet denoising across various decomposition levels at an *SNR* of 15 dB.

Decomposition Level	Jarque–Bera Test	True Value Known		True Value Unknown		*F*	*T*	*H*
		*RMSE*	*SNR*	*RMSE*	*r*			
2	accept	0.2360	21.2025	0.3926	0.0469	0.1944	0.2007	0.1983
3	accept	0.1757	23.7674	0.4231	0.0214	0.1010	0.1245	0.1157
4	accept	0.1531	24.9619	0.4497	0.0175	0.0950	0.1270	0.1150
5	accept	0.2962	19.2315	0.5223	0.0107	0.0947	0.1486	0.1285
6	accept	0.3699	17.3007	0.5614	0.0072	0.0956	0.1612	0.1367
7	accept	0.3988	16.6466	0.5793	0.0070	0.1018	0.1722	0.1459
8	accept	0.4044	16.5261	0.5865	0.0070	0.1042	0.1766	0.1496
9	accept	0.4199	16.2001	0.5959	0.0069	0.1075	0.1824	0.1544
10	accept	0.4257	16.0799	0.6028	0.0069	0.1102	0.1869	0.1582

**Table 5:** Optimal decomposition levels under different *SNR* conditions (using the db4 wavelet basis as an example).

*SNR* (dB)	True Value Known	True Value Unknown		
		*F*	*T*	*H*
−3	4	4	4	4
3	4	4	4	4
9	4	4	3	4
12	4	4	3	4
15	4	5	3	4
18	3	3	3	3

**Table 6:** Optimal decomposition levels under different wavelet basis functions (using 15 dB *SNR* simulation data as an example).

Wavelet Basis Function	True Value Known	True Value Unknown		
		*F*	*T*	*H*
db4	4	5	3	4
db5	4	4	3	4
sym5	4	4	3	4
sym6	4	4	3	4
coif4	4	4	4	4
coif5	4	5	4	4

**Table 7:** Analysis of the comparison of comprehensive indicators for different decomposition levels.

Decomposition Level	Jarque–Bera Test	*F*	*T*	*H*
1	accept	0.8773	0.8251	0.8433
2	accept	0.1999	0.2020	0.2012
3	accept	0.0952	0.1109	0.1054
4	accept	0.0889	0.1113	0.1035
5	accept	0.1001	0.1338	0.1220
6	accept	0.1067	0.1495	0.1345
7	accept	0.1133	0.1610	0.1443
8	accept	0.1179	0.1679	0.1504
9	accept	0.1203	0.1715	0.1536
10	accept	0.1215	0.1732	0.1552
11	accept	0.1227	0.1749	0.1567

**Table 8:** Different decomposition levels and their corresponding corrected *RMSE*.

Decomposition Level	2	3	4	5	6	7	8	9	10	11
*RMSE*	0.2134	0.2105	0.2097	0.2139	0.2151	0.2183	0.2195	0.2213	0.2233	0.2251

## Data Availability

The original contributions presented in the study are included in the article, and further inquiries can be directed to the corresponding author.
